# MGRA: Motion Gesture Recognition via Accelerometer

**DOI:** 10.3390/s16040530

**Published:** 2016-04-13

**Authors:** Feng Hong, Shujuan You, Meiyu Wei, Yongtuo Zhang, Zhongwen Guo

**Affiliations:** 1 Department of Computer Science and Technology, Ocean University of China, Qingdao 266100, China; youshujuan@gmail.com (S.Y.); weimeiyu90@163.com (M.W.); guozhw@ouc.edu.cn (Z.G.); 2 School of Computer Science and Engineering, The University of New South Wales, Sydney 2052, Australia; ytzhang@cse.unsw.edu.au

**Keywords:** accelerometer, gesture recognition, SVM, feature selection

## Abstract

Accelerometers have been widely embedded in most current mobile devices, enabling easy and intuitive operations. This paper proposes a Motion Gesture Recognition system (MGRA) based on accelerometer data only, which is entirely implemented on mobile devices and can provide users with real-time interactions. A robust and unique feature set is enumerated through the time domain, the frequency domain and singular value decomposition analysis using our motion gesture set containing 11,110 traces. The best feature vector for classification is selected, taking both static and mobile scenarios into consideration. MGRA exploits support vector machine as the classifier with the best feature vector. Evaluations confirm that MGRA can accommodate a broad set of gesture variations within each class, including execution time, amplitude and non-gestural movement. Extensive evaluations confirm that MGRA achieves higher accuracy under both static and mobile scenarios and costs less computation time and energy on an LG Nexus 5 than previous methods.

## 1. Introduction

The Micro-electromechanical Systems (MEMS) based accelerometer is one of the most commonly-used sensors for users to capture the posture, as well as the motion of devices [1]. Extensive research has been carried out based on the accelerometer data of mobile devices, including phone placement recognition [2], knee joint angle measurement [3], indoor tracking [4] and physical activity recognition [5,6]. Research conducted so far still faces a challenging problem, which is not tackled effectively: signal drift or the intrinsic noise of MEMS-based accelerometers on commercial mobile devices.

Moreover, the accelerometer enables a mobile device to “sense” how it is physically manipulated by the user. As a result, a new type of interaction based on motion gestures performed by the users has been proposed, making eye-free interactions possible without stopping movement. The objective of a motion gesture recognition system is to find out which gesture is intended by the users, which is a spatio-temporal pattern recognition problem. Except the problem of accelerometer signal drift or intrinsic noise, motion gesture recognition systems confront three new challenges as follows:

- Intuitively, one cannot promise to perform the same gesture exactly twice. The motion gestures usually vary strongly in execution time and amplitude. Therefore, the gesture recognition system should take into account the motion variances of the users.
- The gesture recognition system should provide on-the-move interaction under certain mobile scenarios, like driving a car or jogging. The non-gestural user movements have an effect on acceleration signals, making gesture recognition more difficult.
- Training and classification of the motion gestures are expected to be executed entirely on the mobile devices. Therefore, the computation and energy costs need to be limited for such self-contained recognition systems.

Previous work on motion gesture recognition can be categorized into two types: template-based and model-based. Template-based approaches store some reference gestures beforehand for each class and match the test gesture with some similarity measurements, such as Euclidean distance [7]. uWave [8] applies Dynamic Time Warping (DTW) to evaluate the best alignment between gesture traces in order to tackle execution time variation. Model-based methods are based on the probabilistic interpretation of observations. Exploiting Hidden Markov Model (HMM), 6DMG [9] is generally robust to time and amplitude variations. The recognition accuracy of previous research, however, is somehow affected by non-gestural user movements, like sitting in a running vehicle, which will be shown in the Evaluation section. Furthermore, most previous works carry out the calculation on a nearby server instead of on mobile devices, which may involve privacy issues.

In order to solve all of the above issues, we try to answer one non-trivial question: *what are the robust and unique features for gesture recognition hidden in the raw acceleration data?* This paper is dedicated to extracting robust features from the raw acceleration data and exploits them to realize gesture recognition on mobile devices. The features should accommodate a broad set of gesture variations within each class, including execution time, amplitude and non-gestural motion (under certain mobile scenarios).

In our solution, we first collected 11,110 motion gesture traces on 13 gestures performed by eight subjects across four weeks, among which, 2108 traces are collected under mobile scenarios. We then enumerate the feature set based on the time domain, the frequency domain and Singular Value Decomposition (SVD) analysis. The best feature vector of 27 items is selected under the guide of mRMR [10], taking both static and mobile scenarios into consideration. We then implement our Motion Gesture Recognition system using Accelerometer data (MGRA) with the best feature vector, exploiting SVM as the classifier. The implementation is on an LG Nexus 5 smartphone for the evaluations. MGRA is first evaluated through off-line analysis on 11,110 motion traces, comparing accuracy with uWave [8] and 6DMG [9]. The results demonstrate that MGRA achieves an average accuracy of 95.83% under static scenarios and 89.92% under mobile scenarios, both better than uWave and 6DMG. The computation and energy cost comparison on the LG Nexus 5 also confirms that MGRA outperforms uWave and 6DMG.

The major contributions are as follows:

- A comprehensive gesture set of 11,110 motion traces was collected containing 13 gestures performed by eight subjects across four weeks, among which, 2108 traces are collected under mobile scenarios. Based on this dataset, 34 statistical features are enumerated through the time domain, the frequency domain and SVD analysis with the visualization of their impact on gesture classification.
- We exploit mRMR to determine the feature impact order on gesture classification for static and mobile scenarios, respectively. The best feature vector of 27 items is empirically chosen as the intersection of these two orders.
- The MGRA prototype is implemented with the best feature vector on the LG Nexus 5. We compare MGRA on classification accuracy, computation and energy cost under both static and mobile scenarios to previous research of uWave and 6DMG. MGRA achieves the best performance on all metrics under both scenarios.

The rest of this paper is organized as follows. In Section 2, we introduce the technical background on motion gesture recognition. Section 3 illustrates our data collection process and our observation on execution time, amplitude and scenario variations based on our gesture sets. Details on feature enumeration are described in Section 4, and Section 5 presents the feature selection process. Section 6 gives a system overview of MGRA. Section 7 presents the comparison of the accuracy of MGRA to uWave and 6DMG on two gesture sets, both under static and mobile scenarios. It also shows the time and energy cost of MGRA, uWave and 6DMG on Android smartphones. We conclude our work in Section 8.

## 2. Related Work

This section reviews the research efforts on gesture recognition systems based on the accelerometer for mobile devices. The objective of a gesture recognition system is to classify the test gesture (that the user just performed) to a certain class according to the training gesture set (that the user performed early).

Previous research can be mainly categorized into two types: template-based and model-based. Intuitively, some basic methods measure the distance between the test gesture and the template gestures of each class and select the class with the minimum distance as the result. Rubine [11] made use of the geometric distance measure on single-stroke gestures. Wobrock *et al.* [7] exploited the Euclidean distance measurement after uniformly resampling the test gesture to handle execution time variation.

To cope with sampling time variations, several methods based on Dynamic Time Warping (DTW) are presented. A similarity matrix is computed between the test gesture and the reference template with the optimal path, representing the best alignments between two series. Wilson *et al.* [12] applied DTW on the raw samples from the accelerometer and gyroscope for gesture recognition. uWave [8] first quantized the raw acceleration series into discrete values, then employed DTW for recognition. Akl and Valaee [13] exploited DTW after applying compressive sensing on raw accelerations. Nevertheless, the amplitude variation still affects the recognition accuracy for the aforementioned DTW-based methods.

Statistical methods, such as the widely-used Hidden Markov Model (HMM), are based on probabilistic interpretation of gesture samples to model the gestural temporal trajectory. HMM-based methods are generally robust, as they rely on learning procedures on a large database, creating a model accommodating variations within a gesture class. Each underlying state of HMM has a particular kinematic meaning and describes a subset of this pattern, *i.e.*, a segment of the motion. Schlömer *et al.* [14] leveraged the filtered raw data from the acceleration sensor embedded in the Wii remote and evaluated 5, 8 and 10 states, respectively, for motion model training. 6DMG [9] extracted 41 time domain features from the samples of accelerometer and gyroscope of the Wii remote. It exploited eight hidden states for building an HMM model with 10 training traces for each class.

Support Vector Machine (SVM) is also extensively applied for motion gesture recognition. SVM-based methods usually offer lower computational requirements at classification time, making them preferable for real-time applications on mobile devices. The gesture classification accuracy depends closely on the feature vector for SVM-based methods. Wu *et al.* [15] extracted the feature of mean, energy and entropy in the frequency domain and the standard deviation of the amplitude, as well as the correlation among three axes in the time domain. As the raw time series are divided into nine segments and each feature is repeatedly extracted from every segment, the total feature set contains 135 items in [15]. In [16], Haar transform was adopted in the feature extraction phase and produces descriptors for modelling accelerometer data. The feature set contains 24 items.

In this paper, we also exploit SVM as the core of MGRA owing to its low computation cost on classification. Different from previous approaches, we focus on feature enumeration through not only the time domain and the frequency domain, but also SVD analysis. Then, we select the feature vector of 27 items based on mRMR, taking both static and mobile scenarios into consideration. We realize MGRA entirely on an Android smartphone, the LG Nexus 5.

## 3. Gesture Design and Data Collection

This section introduces our gesture collection phase and reveals some key observations on raw sampling data of motion gestures.

### 3.1. Gesture Collection

We developed a motion gesture collection application on the LG Nexus 5 with a sampling rate of 80 Hz. To make the users free from interactions with the touch screen, we rewrote the function of the short press on the power button. In our approach, the application records the accelerometer readings after the first press on the power button and ends on the second press. During the interval of two presses, the users perform motion gestures.

Eight subjects, including undergraduates, graduates and faculty members, participated in data collection, with ages ranging from 21 to 37 (ethical approval for carrying out this experiment has been granted by the corresponding organization). Each subject was asked to perform gestures in his or her own convenient style. We did not constrain her or his gripping posture of the phone, the scale and the speed of the action. The subjects only participate when available. We asked them to perform each gesture no less than 20 times for one collection period.

**Figure 1:**
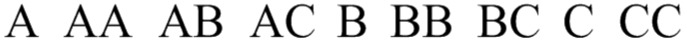
Gestures used in Confusion Set.

Since a necessary and sufficient number of single gestures is needed for phone command control, we define nine gestures as our classification target. These nine gestures are chosen as the combination of upper letters “*A*”, “*B*” and “*C*”, as shown in Figure 1. The choice of these gestures is due to two reasons, as follows:

(1) Each gesture shares one character with the other three gestures, ensuring the difficulty of recognition. If these gestures can be classified with high accuracy, the gestures of other characters’ combination customized by future end users will keep high recognition precision.

(2) The choice of spatial two-dimensional symbol gestures is consistent with the previous survey study [17]. Our survey on motion gesture design from 101 freshmen also indicates that there are 96.8% of 218 gestures created as combinations of English characters, Chinese characters and digits. The statistics of the designed gestures is shown in Table 1.

**Figure 2:**
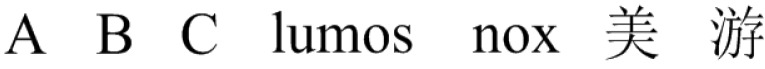
Gestures used in Easy Set.

We further collected two English words “lumos” and “nox” (magic spells from the *Harry Potter* novel series) and two Chinese characters to build the second gesture set. The gestures in this set share no common parts with each other; shown in Figure 2. We name the gesture set in Figure 1 Confusion Set and the second in Figure 2 Easy Set. We thereby construct MGRA targeted on Confusion Set and verify the recognition results with Easy Set to prove the gesture scalability of MGRA in the Evaluation section. Taking the mobile situations into consideration, our subjects collect the gesture traces not only under static scenarios, but also sitting in a running car. After four weeks, we collected 11,110 gesture traces, among which 2108 are performed under mobile scenarios. The amounts of all kinds of bespoken gestures are summarized in Table 2.

### 3.2. Observation on Gesture Traces

A motion gesture trace is described as a time series of the acceleration measurements. $a=(a_{1},a_{2},...,a_{n})$, where $a_{i}={\left[a_{x}\left(i\right),a_{y}\left(i\right),a_{z}\left(i\right)\right]}^{T}$, is a vector of *x*, *y* and *z* acceleration components according to the phone axes. *n* is the number of samples within the whole gesture.

**Figure 3:**
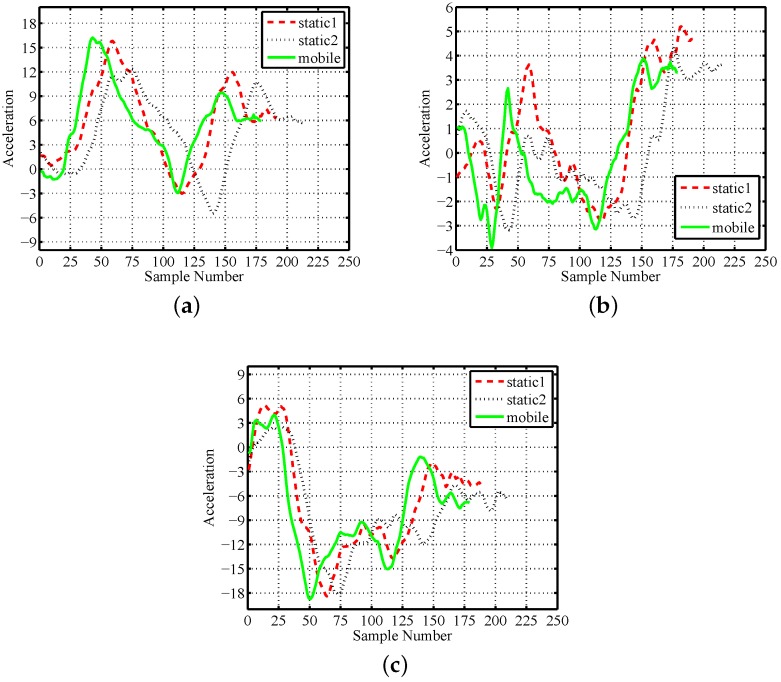
Motion traces of gesture *A* by one subject: (**a**) X-axis; (**b**) Y-axis; (**c**) Z-axis.

Figure 3 shows the raw acceleration series of three typical motion traces of gesture *A* performed by one subject. The first and second traces are chosen from static traces, where the third is selected from mobile traces. It is observable that the motion patterns usually vary strongly on execution time, amplitude and scenario type. For example, the difference on execution time is about 0.5 s between two static gestures. The maximum value on the X-axis of one static trace is about three higher than the other in Figure 3a. Moreover, the values of the mobile trace are commonly larger than the two static traces on all three axes. The same phenomena coincide with other subjects. We deem these phenomena as the time, amplitude and scenario variety of motion gestures and discuss them in detail in the following subsections.

#### 3.2.1. Time Variety

**Figure 4:**
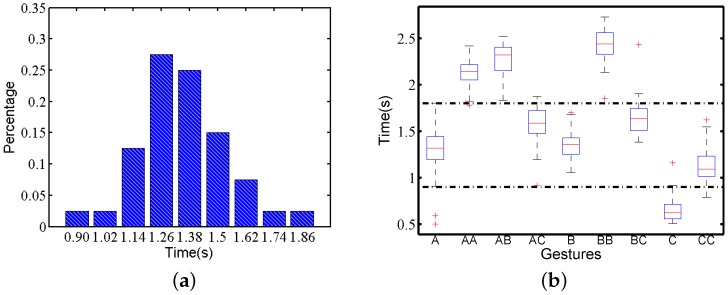
Execution time on gesture *A* by one subject: (**a**) distribution; (**b**) box plot.

We count the execution time of all motion gesture traces in Confusion Set. Figure 4a shows the execution time distribution for one subject performing gesture *A*, which is similar to a Gaussian distribution. This is reasonable because a person normally cannot control actions precisely under the unit of seconds, while the whole gestures are finished in no more than 2 s. Figure 4b presents the box plot of the gesture traces in Confusion Set performed by the same subject. It shows that execution time variety exists among all gestures. We divide these nine gestures into three groups according to the execution time. The groups of gestures AA, AB and BB have a longer execution time, while gesture *C* has a shorter execution time. The remaining forms the third group. Therefore, applying just the time length can distinguish three gesture groups for this subject. This conclusion is consistent with the motion traces of other subjects.

#### 3.2.2. Amplitude Variety

**Figure 5:**
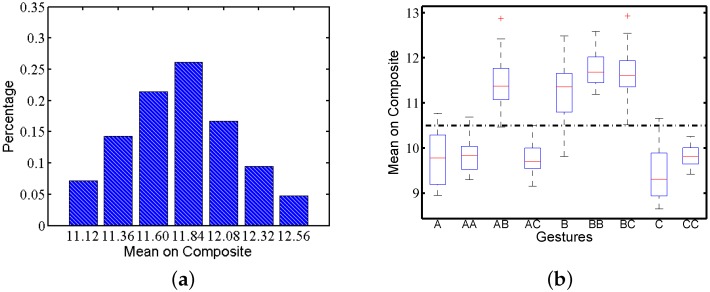
Mean composite acceleration of gesture AB by one subject under static scenarios: (**a**) distribution; (**b**) box plot.

We calculate the composite acceleration of the raw traces to estimate the whole strength when a user is performing gestures. The composite acceleration of the raw traces comes from Equation (1), which captures the user behaviour as a whole. The mean composite acceleration of the gesture indicates the strength used to perform the gesture by subjects. Figure 5a shows the average composite acceleration distribution of gesture AB performed by one subject under static scenarios. Similar to time variation, the distribution is also like a Gaussian distribution. Figure 5b shows the box plot of the mean composite acceleration amplitude among all motion traces. There is amplitude variety for all gestures performed by this subject, even under only static scenarios. According to Figure 5b, nine gestures can be categorized into two groups: gestures AB, *B*, BB and BC have a relatively higher strength than the other gestures. For other subjects, the variety in amplitude also exists.

(1) $$a_{c}\left(i\right)=\sqrt{{a_{x}\left(i\right)}^{2}+{a_{y}\left(i\right)}^{2}+{a_{z}\left(i\right)}^{2}}$$

#### 3.2.3. Scenario Variety

**Figure 6:**
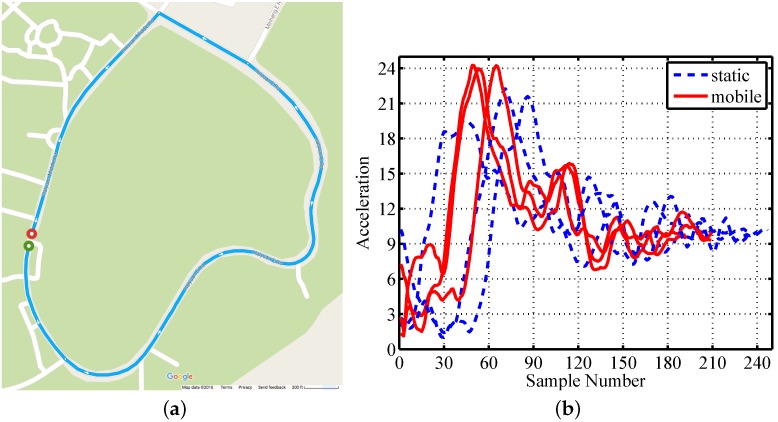
Mobile trace collecting: (**a**) car trajectory; (**b**) examples under static and mobile scenarios.

There is a wide variety of mobile devices that have no common features, except mobility. We collect the gesture traces in a running car as an example for mobile scenarios. We drive the car in the university campus to collect mobile traces, and the trajectory is shown in Figure 6a. We then compare the composite accelerations under mobile scenarios to the values under static scenarios, shown in Figure 6b. The amplitude of composite acceleration is higher under mobile scenarios than that under static scenarios. This is mainly because the car contributes to acceleration values when changing speed.

Time, amplitude and scenario varieties have been observed from our gesture set, which can have a direct impact on the recognition accuracy. Hence, we think about extracting the robust and unique features from the raw acceleration series for gesture recognition, which can help in accommodating these three varieties.

## 4. Feature Enumeration

Feature extraction is the fundamental problem in the area of pattern recognition. However, few works, which extract effective features and make a quantitative comparison of their quality for gesture recognition, have been reported. This section illustrates our feature enumeration from acceleration data through the time domain, the frequency domain and SVD analysis.

### 4.1. Time Domain Features

The motion gestures are different from each other in their temporal spatial trajectories. The trajectory can be reflected by time domain features to some degree. We extract the time domain features from the raw acceleration traces.

As discussed in Section 3.2.1, the gestures can be classified into three groups based only on execution time length. Therefore, we take the time length as our first feature, labelled as $\left\{f_{1}\right\}$.

**Figure 7:**
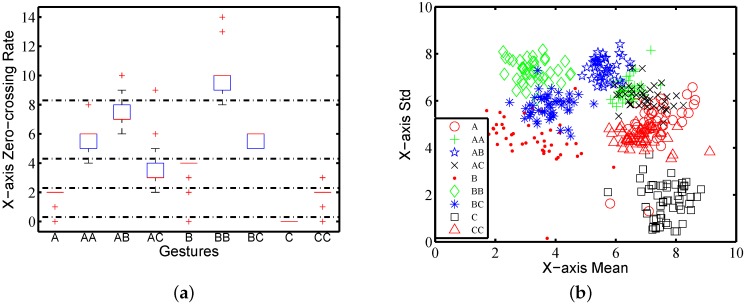
Features’ impact on gesture classification: (**a**) zero-crossing rate; (**b**) mean and standard deviation (std) on the X-axis.

Due to the difference in spatial trajectories, the number of turns in an action may be used to distinguish different gestures. We use zero-crossing rates on three columns of raw traces to estimate the change of acceleration, reflecting some clues about the spatial trajectories. Figure 7a shows the zero-crossing rate on the X-axis of nine gestures performed by one subject. It shows that nine gestures can be categorized into five groups according to the zero-crossing rate on the X-axis, which are the groups {C}, {A,CC}, {AC,B}, {AA,AB,BC} and {BB}. We thereby treat the zero-crossing rate on three axes as feature $\left\{f_{2},f_{3},f_{4}\right\}$.

The composite acceleration of the raw traces calculated as Equation (1) can capture the user behaviour as a whole. Here, we calculate the mean and standard deviation (std) on composite acceleration, as well as each column. The mean shows how much strength the user uses when performing certain gestures. The standard deviation reveals how the user controls his or her strength during performing a gesture.

Figure 7b shows the results of the mean and standard deviation on the X-axis for nine gestures performed by one subject. Each gesture class contains 40 traces. The points of the same gestures are clustered, indicating that different gestures performed by the same subject have different strength averages and variations. For example, the traces of gestures AB, *B*, BB, *C* and CC are clearly separated in Figure 7b. The results from data on other axes and on composite accelerations are empirically similar. This confirms that the mean and standard deviation can to some extent work in gesture classification. Hence, we label the eight items of the mean and standard deviation as $\left\{f_{5},f_{6},...,f_{12}\right\}$.

**Figure 8:**
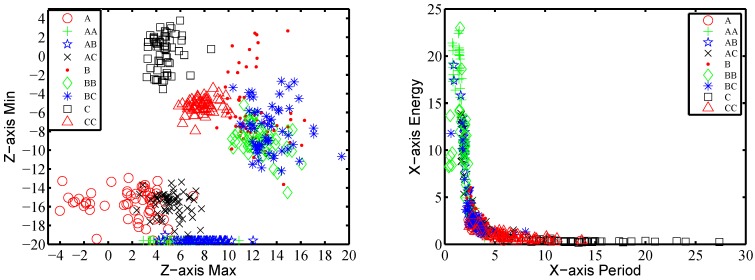
Features’ impact on gestures classification: (**a**) maximum and minimum in the Z-axis; (**b**) period and power in the X-axis of a certain frequency.

We further calculate the maximal and minimal values on three axes, respectively, and on composite acceleration. Figure 8a shows the maximal and minimal acceleration on the Z-axis for nine gestures performed by one subject. Each class contains 40 trials. It provides some boundaries for gesture recognition, e.g., Figure 8a shows that the traces of AC, BC and *C* can be distinguished by these two parameters. Therefore, we take these eight values as features $\left\{f_{13},f_{14},...,f_{20}\right\}$. The time complexity for extracting time domain features turns out to be O(n).

### 4.2. Frequency Domain Features

We extract the features from the frequency domain. We apply Fast Fourier Transform (FFT) on the three columns of the raw traces. Previous research took all of the low frequency parts directly as the features [18], but found the recognition results to be worse than just by calculating the correlation from the original time series.

Unlike previous approaches, we assume that people have an implicit frequency while performing certain gestures, and we try to locate it. We select the frequency with the largest energy instead of the base and second frequency and use it to represent the frequency domain features. The second frequency always has significantly high energy for most gestures containing repeating symbols, e.g., AA. In order to align with the different time lengths of the motion traces, we take the period as the feature instead of the frequency. Therefore, the frequency feature includes the period and energy of a certain frequency.

Figure 8b shows the frequency feature on the X-axis of 40 traces per gesture performed by one subject. It shows that some pairs or groups of gestures can be distinguished by these two parameters. For example, the traces of gestures AA, BC and *C* have no intersection among one another in Figure 8b. Similar results occur in the other axes practically. Therefore, the period and energy features on the three axes are all adopted into our feature set as $\left\{f_{21},f_{22},...,f_{26}\right\}$, respectively. The time complexity to compute the frequency features is O(nlogn).

### 4.3. SVD Features

During the data collecting phase, we observe that the subjects tend to hold the phone in different postures to perform different gestures. Therefore, we aim to represent such posture differences. Singular Value Decomposition (SVD) provides a unique factorization of the form $A=U\Sigma V^{*}$. In the scenario of motion traces, *n* is the sample number of the traces, *U* is an n×3 unitary matrix, Σ is a 3×3 diagonal matrix with non-negative real numbers on the diagonal and $V^{*}$ denotes the conjugate transpose of a 3×3 unitary matrix. The diagonal entries $\sigma_{i}$ are the singular values of *A* listed in descending order. The complexity of SVD on the motion traces is O(n).

**Figure 9:**
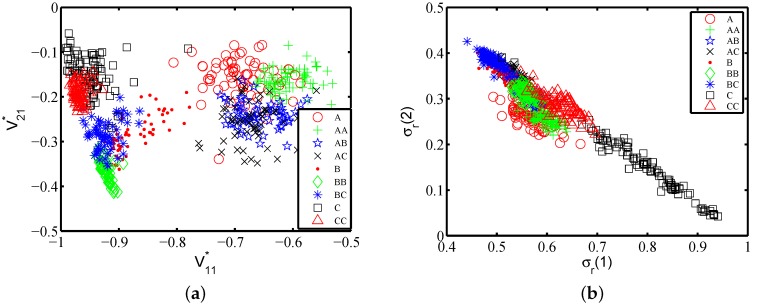
SVD features: (**a**) $V_{11}^{*}$ and $V_{21}^{*}$; (**b**) *σ*.

$V^{*}$ is the rotation matrix from the actual motion frame to the phone frame, indicating the gripping posture. As the gestures that we are studying are 2D gesture, the first and the second column vector of $V^{*}$ can be critical for the phone posture estimation, labelled as $\left\{f_{27},f_{28},...,f_{32}\right\}$. Figure 9a shows $V_{11}^{*}$ and $V_{21}^{*}$ of one subject in which each gesture class contains 40 traces. It shows that the nine gestures can be first divided into two groups on parameter $V_{11}^{*}$, which are groups {A,AA,AB,AC} and {B,BB,BC,C,CC}. This indicates that this subject uses different phone gripping postures when performing the gestures starting with different characters. There is further discriminative power inside each group. For example, gestures BB and CC can be separated on parameter $V_{21}^{*}$.

We then dig into singular value Σ. Σ represents the user’s motion strength on three orthogonal directions when performing actions. Recall that even the same user cannot perform identical gestures with exactly the same strength, and our gesture recognition feature should be suitable under mobile scenarios; so, we leverage the relative value, called the *σ*-rate ($\sigma_{r}$), defined as follows:

(2) $$\sigma_{r}=\left(\frac{\sigma_{1}}{\sigma_{1}+\sigma_{2}+\sigma_{3}},\frac{\sigma_{2}}{\sigma_{1}+\sigma_{2}+\sigma_{3}}\right)$$

The *σ*-rate represents how the user allocates her or his strength on orthogonal directions when performing a gesture relatively. Figure 9b shows features $\sigma_{r}\left(1\right)$ and $\sigma_{r}\left(2\right)$ on 40 traces per gesture performed by one subject. In Figure 9b, gestures BC and *C* are clearly not intersected, showing that the $\sigma_{r}$ can provide some clues to classify different gestures. Therefore, we add $\sigma_{r}$ to the feature set, labelled as $\left\{f_{33},f_{34}\right\}$.

As *U* contains the time series information, which has been extracted by the time domain and frequency domain analysis, we leave *U* out of consideration.

All together, the feature set is composed of 34 features, shown in Table 3. Though we only depict the classification impact of features on one subject, similar results empirically exist for other subjects.

## 5. Feature Selection

Feature selection is another elementary problem for pattern classification systems. We select the best feature vector using the mRMR approach [10] and Confusion Set validation. mRMR determines the feature order that minimizes the redundancy and maximizes the relevance to minimize the classification error.

We first apply mRMR on Confusion Set under static scenarios to get the impact order of the features on gesture classification. The descending order result is $\{f_{12},f_{1},f_{31},f_{33},f_{4},f_{2},f_{28},f_{19},f_{20},f_{5},f_{9},f_{15},f_{10},f_{16},f_{14},f_{7},f_{13},f_{17},f_{11},f_{34},f_{22},f_{8},f_{6},f_{18},f_{32},f_{30},f_{27},f_{29},f_{26},f_{3},f_{24},f_{23},f_{21},f_{25}\}$, labelled as $F_{static}$. Then, we apply mRMR on the mobile traces of Confusion Set, resulting in $\{f_{12},f_{1},f_{31},f_{4},f_{2},f_{28},f_{3},f_{19},f_{20},f_{5},f_{9},f_{33},f_{15},f_{10},f_{16},f_{14},f_{7},f_{13},f_{17},f_{8},f_{22},f_{34},f_{6},f_{18},f_{30},f_{27},f_{32},f_{24},f_{29},f_{26},f_{11},f_{21},f_{25},f_{23}\}$, labelled as $F_{mobile}$. The target feature vector should be suitable for both static and mobile scenarios. We calculate the ordered intersection $F_{in}$ on $F_{static}$ and $F_{mobile}$ according to Equation (3) through Algorithm 1. In the realization of Algorithm 1, we use the linked list for *S* and *M*. When adding an item to *F*, we delete this item in *S* and *M* to speed up the operation of “∈”. In order to make Algorithm 1 easy to understand, we leave out the details of this speed up trick.

(3) $$F_{in}\left(1:x\right)=F_{static}\left(1:x\right)\cap F_{mobile}\left(1:x\right)$$

The result of $F_{in}\left(x\right)$ is thereby shown in Table 4. When executing Algorithm 1, there can be some value of *x* that no new feature is added to *F*. For example, when x=4, the $F_{in}\left(4\right)=null$. This is because $F_{in}\left(1:4\right)=F_{in}\left(1:3\right)=\left\{f_{12},f_{1},f_{31}\right\}$. Meanwhile, $F_{in}\left(x\right)$ may add two features together for some value of *x*, like x=22, as shown in Table 4. As illustrated in Algorithm 1, we add these two features according to their impact order under static scenarios. For x=22, we first add feature $f_{34}$ and add $f_{8}$ afterwards. After coping with the intersection order, we delete the “null” out, and all 34 features are sorted in $F_{in}$.

mRMR only provides the order of $F_{in}$; we further verify the classification results of $F_{in}\left(1:x\right)$ for x=1,2...34 in Section 7.1. Here, we report that the best classification result comes from $F_{in}\left(1:27\right)$ *i.e.*, $\left\{f_{12},f_{1},f_{31},f_{4},f_{2},f_{28},f_{19},f_{20},f_{5},f_{9},f_{33},f_{15},f_{10},f_{16},f_{14},f_{7},f_{13},f_{17},f_{22},f_{34},f_{8},f_{6},f_{18},f_{30},f_{32},f_{27},f_{29}\right\}$. The best feature vector contains 27 items, including time length, zero-crossing rates on the X-axis and the Y-axis, the mean and standard deviation on each column, the standard deviation of composite acceleration, the maximal and minimal values of accelerations on three axes and the composite acceleration, the energy on the X-axis, the first and the second column vector of $V^{*}$ and $\sigma_{r}$. The time complexity to calculate $F_{in}\left(1:27\right)$ turns out to be O(nlogn).


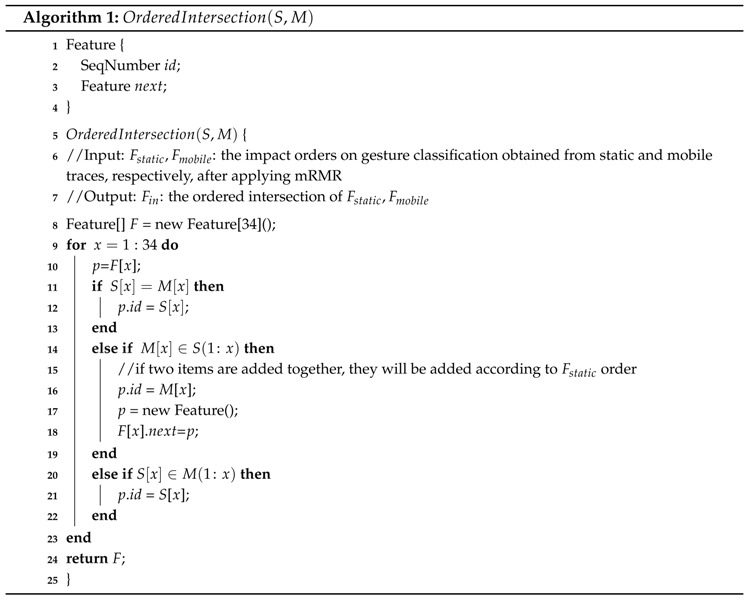


## 6. Design of MGRA

**Figure 10:**
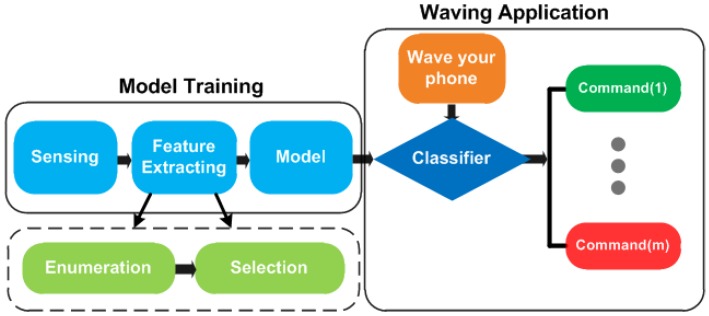
Sketch of the Motion Gesture Recognition system using Accelerometer data (MGRA) design (m referring to the class numbers of gestures).

Due to constrained computing and storage resources and being concerned with time consumption, we use multi-class SVM as the core for gesture recognition. The design of MGRA is shown in Figure 10, including five major components:

- *Sensing*: recording the acceleration data when a user is performing motion gestures between two presses on the phone power button.
- *Feature enumeration*: enumerating the feature set from the raw acceleration series through the time domain, the frequency domain and SVD analysis.
- *Feature selection*: determining the feature impact order on classification through the mRMR method, under static and mobile scenarios separately. The intersection of two orders is exploited to determine the best feature vector according to the classification evaluation on Confusion Set.
- *Model construction*: constructing the multi-class SVM recognition model using the best feature vector through training process.
- *Classifier*: recognizing the input gesture and triggering a certain phone command for the users.

The kernel of SVM exploits RBF, in which the parameters (c,γ) are tuned through five-fold cross-validation on the training data. MGRA only needs 10 traces per motion gesture class to train the model. The training set number of 10 per class is determined in the next section.

## 7. Evaluation

This section presents the results of the off-line analysis on the collected trace set and the online evaluation on LG Nexus 5 smartphones. For off-line analysis, we first determine the key parameters of MGRA, *i.e.*, the feature vector and the training set number. We further compare two different SVM kernels and one different classifier of random forest to confirm the functionality of SVM with the RBF kernel. Then, we compare the classification accuracy of MGRA with two state-of-the-art methods: uWave [8] and 6DMG [9]. For online evaluation, we compare the energy and computation time among MGRA, uWave and 6DMG.

### 7.1. Parameter Customization

Feature vector selection has a great impact on classification for SVM. Section 5 only provides the feature impact order as $F_{in}$. It still needs to allocate the length of the feature vector; meanwhile, a larger training trace number, better classification accuracy. However, it brings the end users a heavy burden to construct a training set when the number of traces is big.

Hence, we conduct a grid search for optimal values on these two parameters for the static traces, with the feature number $n_{f}$ varying from one to 34 according to the order $F_{in}$ and the training set number $n_{t}$ varying from one to 20. For each combination of these two parameters, we train the SVM model with $n_{t}$ traces per gesture class, randomly selected from Confusion Set under static scenarios for each subject. We only use static traces for training, as the end user may prefer to use MGRA under mobile scenarios, but may not like to collect training traces while moving. After constructing the model, the rest of the traces of each subject under static scenarios is used to test the subject’s own classification model. This evaluation process is repeated five times for each combination.

**Figure 11:**
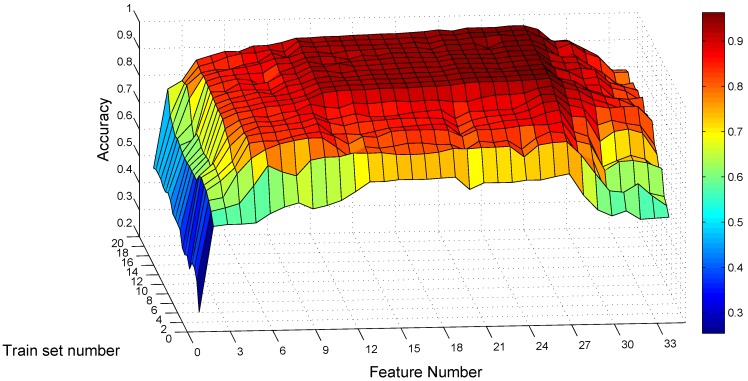
Average recognition accuracy on static traces *vs*. feature number and training set number.

Figure 11 shows the average recognition accuracy of static traces among eight subjects for each parameter combination. From the point of view of the training traces number, it confirms the tendency that a larger number means better classification accuracy. When the training trace number exceeds 10, the accuracy improvement is little. The maximum recognition accuracy under static scenarios is 96.24% with 20 training traces per gesture class and with 27 feature items. For the combination of 10 training traces and 27 features, the average recognition accuracy is 95.83%, no more than 0.5% lower than the maximum value. Hence, we use 10 as the trace number per gesture class for training in MGRA.

We further dig into the impact on recognition with different feature numbers while keeping $n_{t}=10$. Table 5 shows the confusion matrices of one subject on feature number $n_{f}=1,4,11,27$. When only using the standard deviation of the composite acceleration ($f_{12}$), the average classification accuracy is only 44.82%. For example, Table 5a shows that 55% traces of gesture AA are recognized as gesture AB, and the other 45% are recognized as gesture BB. After adding time feature ($f_{1}$), posture $V_{22}^{*}$ ($f_{31}$) and $\sigma_{r}\left(1\right)$ ($f_{33}$), only 7.5% and 5% of gesture AA are recognized incorrectly as gestures AB and BB, described in Table 5b. The average accuracy for four features increases to 83.31%.

After adding seven features, Table 5c shows that there is only 2.5% of gesture AA classified as gesture AB. None of gesture AA is recognized as gesture BB. Such an experiment shows that MGRA with 11 features correctly recognizes between AA and BB. It also illustrates that gesture AA is more confusable with AB than BB, confirming that two gestures are harder to classify when sharing common parts. The average recognition accuracy is 92.52% for 11 features.

For 27 features, the recognition error is little for each gesture class, as shown in Table 5d. Besides, all error items are on the intersection of two gestures sharing a common symbol, except only 2.4% of gesture *A* is recognized incorrectly as gesture *B*. The average recognition accuracy achieves 96.16% for this subject on the feature vector of 27 items.

Before determining the feature number, we should also consider the traces collected under mobile scenarios where the acceleration of the car is added. For each parameter combination, we test the models constructed from the static traces, with the traces collected under mobile scenarios. Someone may ask: why not construct the SVM model based on mobile traces separately? Because it is inconvenient for potential users in practice to collect a number of gestures for training under mobile scenarios. Even collecting the training traces in a car for our subjects, it requires someone else to be the driver. However, it is easy for the user to just perform a certain gesture to call commands on the smartphone under mobile scenarios, comparing to the interaction with touch screens.

**Figure 12:**
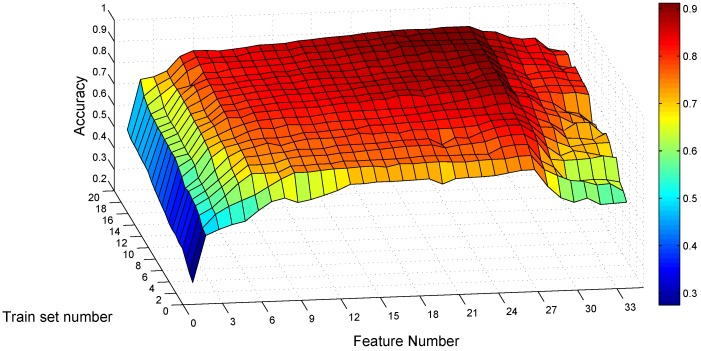
Average recognition accuracy on mobile traces *vs* feature number and training set number.

Figure 12 depicts the classification results for the same subject as Figure 11, testing his static models with mobile traces. The highest accuracy of 91.34% appears when the training set number is 20 and the feature number is 27. For the combination of $n_{t}=10$ and $n_{f}=27$, the recognition accuracy is 89.92%. The confusion matrix of $n_{t}=10$ and $n_{f}=27$ tested with mobile traces is shown in Table 6. Most error items are also on the intersections of gestures sharing one common symbol, except that a small fraction of gesture *B* is classified as gesture *A* and CC, gesture BB as AA and gestures *C* and CC as *B*. Comparing Table 6 to Table 5d, most error cells in Table 6 do not exist in Table 5d. This indicates that the accuracy decreases when testing the classification model constructed from static traces with mobile traces. However, the average recognition accuracy is 90.04%, still acceptable for this subject.

**Figure 13:**
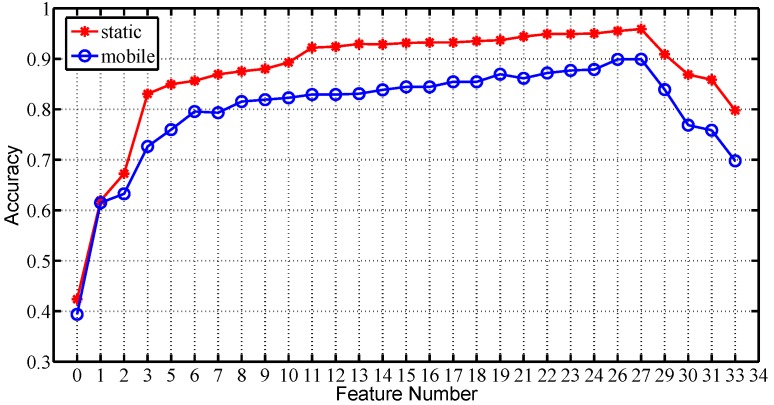
Average recognition accuracy on feature number for both static and mobile scenarios with $n_{t}$ = 10.

Taking the training sample number as 10, we further plot the average recognition accuracy among all subjects for all feature number values under two scenarios respectively, in Figure 13. It shows that the recognition accuracy achieves the maximum when $n_{f}=27$, for the test traces under both static and mobile scenarios. Therefore, we choose $F_{in}\left(1:27\right)$ as the feature vector of MGRA.

### 7.2. Comparison with SVM Kernels and Random Forest

We choose RBF as the SVM kernel for MGRA, withthe assumption based on the central limit theorem. To justify the choice of SVM kernel, we further examine Fisher and polynomial kernels under both static and mobile scenarios. The feature vector is chosen as $F_{in}\left(1:27\right)$ for the comparison.

Besides, some previous research has demonstrated the classification performance of random forest on activity or gesture recognition [19]. Therefore, we also evaluate random forest as the classifier and apply all 34 features under both scenarios.

Table 7 shows the confusion matrix of the Fisher kernel, the polynomial kernel and random forest under static scenarios for the same subject with the results in Table 5d. It first shows that most error items are on the intersections of gestures sharing one common symbol, which is consistent with the error distribution in Table 5d. Table 7a also demonstrates that the Fisher kernel achieves 100% accuracy on classifying repetition gestures, which are AA, BB and CC. However, the accuracy for all of the other gestures is lower than that in Table 5d.

Comparing the polynomial to the RBF kernel, it shows that the classification accuracy of gestures *A*, AA, AB, BB, BC and *C* is higher than 95% in both Table 5d and Table 7b. The misclassification between gestures AC and *B* is larger than 7% in Table 7b for the polynomial kernel, which has been corrected in Table 5d by the RBF kernel. The average classification accuracy among all subjects when applying three different SVM kernels under static scenarios is shown in Table 8. The accuracy of the RBF kernel is the highest, and the other two are close to or above 90% for static traces.

However, the accuracy decreases clearly when applying the model trained under static scenarios to the traces under mobile scenarios for the Fisher and the polynomial kernel, as shown in Table 9a,b. For gestures BC and *B*, the classification accuracy is no more than 60% for both the Fisher and the polynomial kernels. Gesture BC is misclassified as AC and *B* with a high percentage for both kernels, because these three gestures share some common parts. For gesture *B*, there are 27.5% and 32.5% misclassified as gesture CC for both kernels, respectively. These errors come from both gestures being performed as two circles by the subject. The only difference is that gesture *B* includes two vertical circles, while gesture CC contains two horizontal circles. Referring back to Table 6, the RBF kernel misclassifies only 2.5% of gesture *B* as CC under mobile scenarios. The average classification accuracy results among all subjects with three kernels under mobile scenarios is also listed in Table 8. It shows that the Fisher and the polynomial kernel are not robust to scenario change. On the contrary, the RBF kernel retains accuracy very close to 90%.

**Figure 14:**
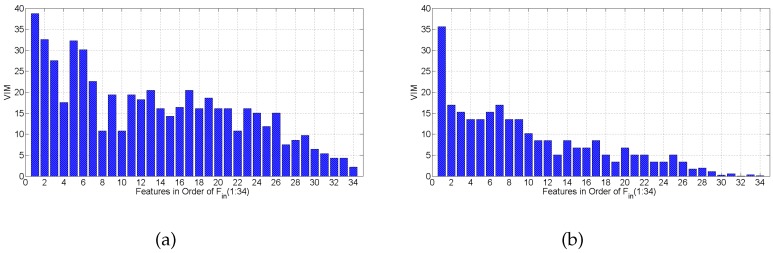
Variable Importance Measure of random forest under different scenarios: (**a**) static; (**b**) mobile.

Applying random forest as the classifier, we get the confusion matrices for the same subject, listed in Table 7c and Table 9c, under both scenarios, respectively. The results show that random forest is also not robust to scenario change. The average accuracy results are lower under both scenarios, when comparing random forest to SVM with the polynomial and RBF kernels, shown in Table 8. When digging into the Variable Importance Measure (VIM) of the random forest average on all subjects, Figure 14 shows that $F_{in}\left(1:27\right)$ are still the most important features for random forest. Except that $F_{in}\left(28,29\right)$ are a little more important than $F_{in}\left(27\right)$ under static scenarios, and $F_{in}\left(28\right)$ outperforms $F_{in}\left(27\right)$ a bit under mobile scenarios. We further conduct an evaluation of random forest on feature set $F_{in}\left(1:27\right)$. The average classification accuracy is 86.17% and 68.32% under static and mobile scenarios, respectively, decreasing no more than 3.5% compared to the accuracy with all 34 features. This confirms that the feature selection results of mRMR and Algorithm 1 are independent of classification method and scenario.

After comparison among three SVM kernels and the classifier of random forest, we confirm that SVM with the RBF kernel is suitable as the classifier for MGRA.

### 7.3. Accuracy Comparison with uWave and 6DMG

We compare MGRA, uWave [8] and 6DMG [9] on classification accuracy with both Confusion Set and Easy Set in this section.

uWave exploits DTW as its core and originally only records one gesture trace as the template. The recognition accuracy directly relies on the choice of the template. To be fair for comparison, we let uWave make use of 10 training traces per gesture in two ways. The first method is to carry out template selection from 10 training traces for each gesture class first. This can be treated as the training process for uWave. The template selection criterion is that the target trace has maximum similarity to the other nine traces, *i.e.*, the average distance from the target trace to the other nine traces after applying DTW is minimum. We call this best-uWave. The second method is to compare the test gesture with all 10 traces per gesture class and to calculate the mean distance from the input gesture to nine gesture classes. We call this method 10-uWave. 10-uWave does not have any training process, but it will spend much time on classification.

For 6DMG, we extract 41 time domain features from both acceleration and gyroscope samples in the gesture traces. The number of hidden states is set to eight, experimentally chosen as the best from the range of (2, 10). 6DMG uses 10 traces per gesture class for training.

We first compare the test trace set from static scenarios. Table 10 shows the confusion matrix of best-uWave, 10-uWave and 6DMG of the same subject of Table 5d. Most classification errors of MGRA, comparing Table 5d to Table 10, still exist when applying best-uWave, 10-uWave and 6DMG; except that best-uWave corrects MGRA’s error of 2.4% on gesture *A* recognizing as gesture *B*. 6DMG corrects MGRA from recognizing 2.5% of gesture AB as gesture AA and 7.3% of gesture AC as BC. Both best-uWave and 10-uWave decrease MGRA’s error of 7.3% to 4.9% on recognizing gesture AC as BC. On the contrary, a majority of errors existing for best-uWave, 10-uWave and 6DMG are corrected by MGRA. For example, the confusion cell of gesture CC recognized as BC is 7.5%, 7.5% and 5.0% for best-uWave, 10-uWave and 6DMG, respectively, in Table 10, which are completely corrected by MGRA. The average accuracy of best-uWave, 10-uWave and 6DMG for this subject under the static scenario is 89.79%, 90.35% and 91.44%, respectively, lower than 96.14% of MGRA.

Then, we compare the performance with the test traces by the same subject under mobile scenarios, shown in Table 11. Comparing Table 6 to Table 11, most recognition errors of MGRA still exist for best-uWave, 10-uWave and 6DMG, except a small fraction of errors are decreased. On the contrary, MGRA corrects or decreases most error items in Table 11. For example, the cell of gesture CC recognized as *B* is 23.1%, 23.1% and 5.1%, respectively, for best-uWave, 10-uWave and 6DMG. Referring back to Table 6, MGRA misclassified only 2.6% of gesture CC as *B*. The reason for gesture CC being misrecognized as *B* is the same as why gesture *B* was misrecognized as CC, as discussed in Section 7.2. Therefore, MGRA outperforms best-uWave, 10-uWave and 6DMG clearly under mobile scenarios, whose average recognition accuracy for this subject is 90.04%, 72.50%, 73.3% and 72.72%, respectively.

**Figure 15:**
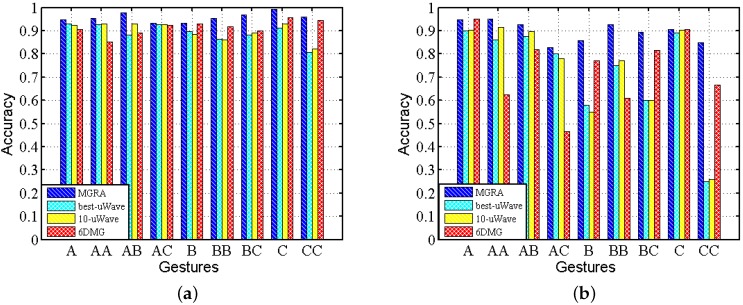
Accuracy comparison among MGRA, best-uWave, 10-uWave and 6DMG on Confusion Set: (**a**) static; (**b**) mobile.

We calculate the average accuracy among all subjects for the test traces under static and mobile scenarios separately and depict the results in Figure 15. It shows that MGRA not only achieves a higher accuracy of classification, but also it has a more stable performance across gestures and scenarios. For best-uWave, 10-uWave and 6DMG, they achieve high accuracy on static traces, but their accuracy decreases about 10% when tested with mobile traces. Moreover, the recognition accuracy is gesture dependent for uWave and 6DMG, especially under mobile scenarios.

Considering the impact of gesture set on recognition, we further compare the recognition accuracy on another gesture set, *i.e.*, Easy Set. The evaluation process is the same as the one on Confusion Set. Table 12 shows the confusion matrices for one subject on Easy Set. Table 12a shows that MGRA achieves higher accuracy on static traces from Easy Set, than the result in Table 12d from Confusion Set. Only 2.5% of gesture *C* is classified incorrectly as *B*, and 5% of gesture 美 is classified incorrectly as 游. The average accuracy is 98.9%. Table 12b shows that MGRA also achieves a higher average accuracy of 95.7% with traces on Easy Set under mobile scenarios. Recall that the features of MGRA are enumerated and selected based totally on Confusion Set. Therefore, the high classification accuracy on Easy Set confirms the gesture scalability of MGRA.

Here, we report the average accuracy comparison among all subjects and all gestures in Table 13. It shows that all approaches improve their accuracy, comparing Easy Set to Confusion Set. For MGRA, the recognition accuracy only decreases 5.48% and 2.87%, from static test traces to mobile test traces for the two gesture sets, respectively. However, the other approaches drop more than 10%. This confirms that MGRA adapts more to mobile scenarios than uWave and 6DMG. Comparing the recognition accuracy on Easy Set, under static and mobile scenarios, MGRA holds accuracy higher than 95% for both. This indicates if the end user puts some efforts into the gesture design, MGRA can achieve high recognition accuracy no matter whether under static or mobile scenarios.

One question might be brought up: why does 6DMG fail in comparison to MGRA, which exploits the readings from both the accelerometer and gyroscope? This result basically is due to two reasons. The first is that MGRA extracts features from the time-domain, the frequency domain and SVD analysis, unlike 6DMG, which only extracts features from the time domain. The second is that MGRA applies mRMRto determine the feature impact order under both static and mobile scenarios and finds the best intersection of two orders. mRMR ensures the classification accuracy for selecting features of the highest relevance to the target class and with minimal redundancy.

### 7.4. Online Evaluation

Energy consumption is one of the major concerns for smartphone applications [20]. Real-time response is also important for user-friendly interaction with mobile devices. Therefore, we conduct a cost comparison for MGRA, best-uWave, 10-uWave and 6DMG on the LG Nexus 5. We measure the energy consumption through PowerTutor [21]. We count the training and classification time for the four recognition methods.

Table 14 shows the cost comparison among the four recognition approaches. MGRA has the smallest time and the minimum energy cost for classification. Moreover, the training time is less than 1 min for MGRA, for it extracts altogether 27 features and is trained with the multi-class SVM model.

The training and classification time is much higher for best-uWave and 10-uWave, because they take DTW on the raw time series as their cores. The raw series contains 200∼400 real values, larger than the 27 items of MGRA. Besides, DTW exploits dynamic programming, whose time complexity is $O(n^{2})$. 10-uWave has a much longer classification time than best-uWave, because the test gesture needs to be compared to all 10 templates for each gesture class, *i.e.*, 90 gesture templates.

The training time and energy of 6DMG is much greater than MGRA, since 6DMG extracts 41 features for the gesture trace and training the HMM model. Besides, the classification time and energy of 6DMG are also greater than those of MGRA. This comes from 6DMG needing both acceleration and gyroscope sensors.

## 8. Conclusions

In this paper, we implement a motion gesture recognition system based only on accelerometer data, called MGRA. We extract 27 features and verify them on 11,110 waving traces by eight subjects. By applying these features, MGRA employs SVM as the classifier and is entirely realized on mobile devices. We conduct extensive experiments to compare MGRA to previous state-of-the-art works, uWave and 6DMG. The results confirm that MGRA outperforms uWave and 6DMG on recognition accuracy, time and energy cost. Moreover, the gesture set scalability evaluation also concludes that MGRA can be applied to both static and mobile scenarios effectively if the gestures are designed to be distinctive.

## Figures and Tables

**Table 1:** Survey Results on user designed motion gestures.

Gesture Type	Percentage
English characters and words	86.7%
Chinese characters	1.4%
digits	8.7%
Graph	3.2%

**Table 2:** Distribution of different gestures in the collection.

Scenarios	A	AA	AB	AC	B	BB	BC	C	CC	Lumos	Nox	美	游	Sum
static	1144	751	745	746	1149	782	752	116	749	209	414	191	204	9002
mobile	229	206	205	213	119	200	201	220	209	123	107	108	117	2108

**Table 3:** Feature set.

$f_{1}$	$f_{2}$–$f_{4}$	$f_{5}$–$f_{10}$	$f_{11}$, $f_{12}$
time	zero-crossing rate	mean and std	mean and std on composite
$f_{13}$–$f_{20}$	$f_{21}$–$f_{26}$	$f_{27}$–$f_{32}$	$f_{33}$, $f_{34}$
maximal and minimal	period and energy	two column vector of $V^{*}$	$\sigma_{r}$

**Table 4:** Ordered feature intersection of $F_{static}\left(1:x\right)$ and $F_{mobile}\left(1:x\right)$.

*x*	1	2	3	4	5	6	7	8	9	10	11	12	13	14	15	16	17	18	19	20	21	22	23	24	25	26	27	28	29	30	31	32	33	34
$F_{static}$	$f_{12}$	$f_{1}$	$f_{31}$	$f_{33}$	$f_{4}$	$f_{2}$	$f_{28}$	$f_{19}$	$f_{20}$	$f_{5}$	$f_{9}$	$f_{15}$	$f_{10}$	$f_{16}$	$f_{14}$	$f_{7}$	$f_{13}$	$f_{17}$	$f_{11}$	$f_{34}$	$f_{22}$	$f_{8}$	$f_{6}$	$f_{18}$	$f_{32}$	$f_{30}$	$f_{27}$	$f_{29}$	$f_{26}$	$f_{3}$	$f_{24}$	$f_{23}$	$f_{21}$	$f_{25}$
$F_{mobile}$	$f_{12}$	$f_{1}$	$f_{31}$	$f_{4}$	$f_{2}$	$f_{28}$	$f_{3}$	$f_{19}$	$f_{20}$	$f_{5}$	$f_{9}$	$f_{33}$	$f_{15}$	$f_{10}$	$f_{16}$	$f_{14}$	$f_{7}$	$f_{13}$	$f_{17}$	$f_{8}$	$f_{22}$	$f_{34}$	$f_{6}$	$f_{18}$	$f_{30}$	$f_{27}$	$f_{32}$	$f_{24}$	$f_{29}$	$f_{26}$	$f_{11}$	$f_{21}$	$f_{25}$	$f_{23}$
$F_{in}$	$f_{12}$	$f_{1}$	$f_{31}$	null	$f_{4}$	$f_{2}$	$f_{28}$	$f_{19}$	$f_{20}$	$f_{5}$	$f_{9}$	$f_{33}$	$f_{15}$	$f_{10}$	$f_{16}$	$f_{14}$	$f_{7}$	$f_{13}$	$f_{17}$	null	$f_{22}$	$f_{34}$,$f_{8}$	$f_{6}$	$f_{18}$	null	$f_{30}$	$f_{32}$,$f_{27}$	null	$f_{29}$	$f_{26}$,$f_{3}$	$f_{11}$,$f_{24}$	null	$f_{21}$	$f_{23}$,$f_{25}$

**Table 5:** Confusion matrices of MGRA on one subject with $n_{t}=10$ and different $n_{f}\left(\%\right)$. (**a**) $n_{f}=1$; (**b**) $n_{f}=4$; (**c**) $n_{f}=11$; (**d**) $n_{f}=27$.

(**a**)									
	A	AA	AB	AC	B	BB	BC	C	CC
A	17.1	9.7	14.6	19.5	0	29.3	9.8	0	0
AA	0	0	55.0	0	0	45.0	0	0	0
AB	0	25.0	75.0	0	0	0	0	0	0
AC	19.5	0	4.8	22.0	0	24.4	19.5	0	9.8
B	0	0	0	0	47.6	0	47.6	0	4.8
BB	12.5	0	37.5	0	0	50.0	0	0	0
BC	10.3	0	0	0	15.4	0	66.7	0	7.7
C	7.5	0	0	0	0	0	0	77.5	15.0
CC	0	0	2.5	10.0	0	15.0	25.0	0	47.5
(**b**)									
	A	AA	AB	AC	B	BB	BC	C	CC
A	75.6	0	0	2.4	4.9	0	0	0	0
AA	0	87.5	7.5	0	0	5.0	0	0	0
AB	0	7.5	92.5	0	0	0	0	0	0
AC	0	0	0	68.3	9.8	0	22.0	0	0
B	0	0	0	0	76.2	0	23.8	0	0
BB	0	0	7.5	0	0	92.5	0	0	0
BC	2.6	0	0	5.1	0	5.1	87.2	0	0
C	2.5	0	0	0	5.0	0	0	92.5	0
CC	0	0	0	5.0	0	0	17.5	0	77.5
(**c**)									
	A	AA	AB	AC	B	BB	BC	C	CC
A	87.8	0	0	7.3	4.9	0	0	0	0
AA	0	97.5	2.5	0	0	0	0	0	0
AB	0	2.5	97.5	0	0	0	0	0	0
AC	0	0	0	85.4	0	0	14.6	0	0
B	0	0	0	0	90.5	0	9.5	0	0
BB	0	0	5.0	0	0	95.0	0	0	0
BC	0	0	0	5.2	0	5.1	89.7	0	0
C	0	0	0	0	5.0	0	0	95.0	0
CC	0	0	0	5.0	0	0	0	0	95.0
(**d**)									
	A	AA	AB	AC	B	BB	BC	C	CC
A	95.2	0	0	2.4	2.4	0	0	0	0
AA	0	97.5	2.5	0	0	0	0	0	0
AB	0	2.5	97.5	0	0	0	0	0	0
AC	0	0	0	92.7	0	0	7.3	0	0
B	0	0	0	0	95.2	0	4.8	0	0
BB	0	0	5.0	0	0	95.0	0	0	0
BC	0	0	0	0	0	2.6	97.4	0	0
C	0	0	0	0	0	0	0	100	0
CC	0	0	0	5.0	0	0	0	0	95.0

**Table 6:** Confusion matrix of MGRA testing static model with mobile traces (%).

	A	AA	AB	AC	B	BB	BC	C	CC
A	95.0	0	0	5.0	0	0	0	0	0
AA	0	95.1	4.9	0	0	0	0	0	0
AB	0	5.0	92.5	2.5	0	0	0	0	0
AC	9.8	0	4.9	82.9	0	0	2.4	0	0
B	5.0	0	0	0	85.0	0	7.5	0	2.5
BB	0	2.5	5.0	0	0	92.5	0	0	0
BC	0	0	0	0	7.3	0	90.2	0	2.4
C	0	0	0	0	5.0	0	0	90.0	5.0
CC	0	0	0	0	2.6	0	10.3	0	87.2

**Table 7:** Confusion matrices of SVM kernels and classification method under static scenarios for one subject. (**a**) Fisher kernel; (**b**) Polynomial kernel; (**c**) Random Forest.

(**a**)									
	A	AA	AB	AC	B	BB	BC	C	CC
A	80.5	0	0	9.8	0	0	0	9.7	0
AA	0	100	0	0	0	0	0	0	0
AB	0	0	92.5	0	0	7.5	0	0	0
AC	0	0	0	90.2	0	0	9.8	0	0
B	7.1	0	0	0	85.8	0	7.1	0	0
BB	0	0	0	0	0	100	0	0	0
BC	0	0	0	12.8	0	0	87.2	0	0
C	0	0	0	7.5	0	0	0	92.5	0
CC	0	0	0	0	0	0	0	0	100
(**b**)									
	A	AA	AB	AC	B	BB	BC	C	CC
A	95.2	0	0	4.8	0	0	0	0	0
AA	0	100	0	0	0	0	0	0	0
AB	0	2.5	95.0	0	0	2.5	0	0	0
AC	7.3	0	0	80.5	7.3	0	0	4.9	0
B	0	0	0	9.4	78.6	0	7.2	0	4.8
BB	0	0	5.0	0	0	95.0	0	0	0
BC	0	0	0	0	0	2.6	97.4	0	0
C	0	0	0	0	0	0	0	100	0
CC	0	0	0	0	0	0	0	10.0	90.0
(**c**)									
	A	AA	AB	AC	B	BB	BC	C	CC
A	75.6	0	0	14.6	9.8	0	0	0	0
AA	0	95.0	5.0	0	0	0	0	0	0
AB	0	2.5	90.0	0	0	2.5	5.0	0	0
AC	0	0	0	87.8	0	0	12.2	0	0
B	0	0	0	0	95.2	0	4.8	0	0
BB	0	0	7.5	0	0	82.5	10.0	0	0
BC	0	0	0	0	0	2.6	97.4	0	0
C	0	0	0	0	0	0	0	100	0
CC	0	0	0	10.0	0	0	0	7.5	82.5

**Table 8:** Accuracy comparison among Fisher, polynomial and random forest testing static models with static and mobile traces, respectively.

Scenarios	RBF	Fisher	Polynomial	Random Forest
Static	95.83%	89.03%	91.42%	89.52%
Mobile	89.92%	72.04%	76.27%	70.87%

**Table 9:** Confusion matrices of SVM kernels and classification method testing static models with mobile traces for one subject. (**a**) Fisher kernel; (**b**) Polynomial kernel; (**c**) Random Forest.

(**a**)									
	A	AA	AB	AC	B	BB	BC	C	CC
A	90.0	0	0	5.0	0	0	0	5.0	0
AA	0	92.6	0	3.7	3.7	0	0	0	0
AB	0	7.5	90.0	0	0	2.5	0	0	0
AC	4.9	0	0	85.4	7.3	0	0	0	2.4
B	7.5	0	0	2.5	60.0	0	0	2.5	27.5
BB	0	0	7.5	0	0	92.5	0	0	0
BC	0	14.6	0	29.4	14.6	0	41.4	0	0
C	0	0	0	0	7.5	0	0	90.0	2.5
CC	0	0	0	5.1	5.1	0	0	25.7	64.1
(**b**)									
	A	AA	AB	AC	B	BB	BC	C	CC
A	95.0	0	0	5.0	0	0	0	0	0
AA	0	95.2	2.4	2.4	0	0	0	0	0
AB	0	5.0	90.0	0	0	5.0	0	0	0
AC	9.8	0	0	73.2	14.6	0	0	0	2.4
B	0	0	0	0	60.0	0	7.5	0	32.5
BB	0	0	15.0	0	0	85.0	0	0	0
BC	0	0	0	31.7	14.6	0	51.2	0	2.5
C	0	0	0	0	2.5	0	0	97.5	0
CC	1.7	0	0	1.7	1.7	0	0	25.6	69.3
(**c**)									
	A	AA	AB	AC	B	BB	BC	C	CC
A	77.5	0	0	17.5	0	0	0	0	0
AA	0	53.6	31.7	14.7	0	0	0	0	0
AB	0	17.5	77.5	5.0	0	0	0	0	0
AC	17.0	0	5.0	78.0	0	0	0	0	0
B	22.5	0	0	0	72.5	0	5.0	0	0
BB	0	0	7.5	5.0	0	80.0	7.5	0	0
BC	0	0	0	12.2	17.1	0	68.3	0	2.4
C	0	0	0	0	7.5	0	0	87.5	5.0
CC	0	0	0	0	10.3	0	10.3	17.9	61.5

**Table 10:** Confusion matrices of three classification methods under static scenarios for one subject. (**a**) best-uWave; (**b**) 10-uWave; (**c**) 6DMG.

(**a**)									
	A	AA	AB	AC	B	BB	BC	C	CC
A	92.7	0	0	7.3	0	0	0	0	0
AA	0	92.5	5.0	0	0	2.5	0	0	0
AB	0	5.0	87.5	0	0	7.5	0	0	0
AC	4.9	0	0	90.2	0	0	4.9	0	0
B	0	0	0	0	90.5	0	9.5	0	0
BB	0	0	10.0	0	0	87.5	0	2.5	0
BC	0	0	0	0	5.1	5.1	87.2	0	0
C	0	0	0	0	2.5	0	0	92.5	5.0
CC	0	0	0	5	0	0	7.5	0	87.5
(**b**)									
	A	AA	AB	AC	B	BB	BC	C	CC
A	90.2	0	0	7.4	2.4	0	0	0	0
AA	0	92.5	5.0	0	0	2.5	0	0	0
AB	0	5	92.5	0	0	2.5	0	0	0
AC	2.4	0	0	92.7	0	0	4.9	0	0
B	0	0	0	0	88.1	2.4	9.5	0	0
BB	0	0	10.0	0	0	87.5	2.5	0	0
BC	0	0	0	0	5.1	5.1	87.2	0	2.6
C	0	0	0	0	2.5	0	0	95.0	2.5
CC	0	0	0	5.0	0	0	7.5	0	87.5
(**c**)									
	A	AA	AB	AC	B	BB	BC	C	CC
A	90.2	0	0	7.3	2.4	0	0	0	0
AA	0	87.5	10.0	2.5	0	0	0	0	0
AB	0	0	90.0	2.5	0	7.5	0	0	0
AC	4.9	0	0	92.7	0	2.4	0	0	0
B	0	0	0	0	92.9	2.4	4.7	0	0
BB	0	0	5.0	0	0	92.5	2.5	0	0
BC	0	0	0	2.6	0	7.7	89.7	0	0
C	0	0	0	0	2.5	0	0	95.0	2.5
CC	0	0	0	2.5	0	0	5.0	0	92.5

**Table 11:** Confusion matrices of three classification methods testing static models with mobile traces for one subject(**a**) best-uWave; (**b**) 10-uWave; (**c**) 6DMG.

(**a**)									
	A	AA	AB	AC	B	BB	BC	C	CC
A	90.0	0	0	7.5	2.5	0	0	0	0
AA	0	85.4	9.7	4.9	0	0	0	0	0
AB	0	10.0	87.5	0	0	2.5	0	0	0
AC	14.6	0	4.9	80.5	0	0	0	0	0
B	12.5	0	0	0	60.0	5.0	22.5	0	0
BB	0	2.5	15.0	0	0	75.0	7.5	0	0
BC	0	0	0	9.7	24.4	4.9	61.0	0	0
C	0	0	0	0	2.5	0	0	90.0	7.5
CC	0	0	0	7.7	23.1	0	30.7	15.4	23.1
(**b**)									
	A	AA	AB	AC	B	BB	BC	C	CC
A	90.0	0	0	7.5	2.5	0	0	0	0
AA	0	90.2	7.4	2.4	0	0	0	0	0
AB	0	7.5	90.0	0	0	2.5	0	0	0
AC	14.6	0	4.9	80.5	0	0	0	0	0
B	12.5	0	0	0	55.0	5.0	27.5	0	0
BB	0	2.5	15.0	0	0	75.0	7.5	0	0
BC	0	0	0	9.7	24.4	4.9	61.0	0	0
C	0	0	0	0	2.5	0	0	95.0	2.5
CC	0	0	0	7.7	23.1	0	30.7	15.4	23.1
(**c**)									
	A	AA	AB	AC	B	BB	BC	C	CC
A	95.0	0	0	5.0	0	0	0	0	0
AA	7.3	61.0	12.2	19.5	0	0	0	0	0
AB	0	7.5	80.0	2.5	0	10.0	0	0	0
AC	19.5	0	9.8	43.9	4.9	0	22.0	0	0
B	12.5	0	0	0	72.5	0	15.0	0	0
BB	0	12.5	20.0	0	0	60.0	7.5	0	0
BC	0	0	0	0	17.1	0	82.9	0	0
C	0	0	0	0	5.0	0	0	92.5	2.5
CC	0	0	0	0	5.1	0	12.8	15.4	66.7

**Table 12:** Confusion matrix of MGRA on Easy Set for one subject. (**a**) test with static traces; (**b**) test with mobile traces.

(**a**)							
	A	B	C	lumos	nox	美	游
A	100	0	0	0	0	0	0
B	0	100	0	0	0	0	0
C	0	2.5	97.5	0	0	0	0
lumos	0	0	0	100	0	0	0
nox	0	0	0	0	100	0	0
美	0	0	0	0	0	95.0	5.0
游	0	0	0	0	0	0	100
(**b**)							
	A	B	C	lumos	nox	美	游
A	94.0	6.0	0	0	0	0	0
B	0	96.0	4.0	0	0	0	0
C	0	6.0	94.0	0	0	0	0
lumos	0	0	0	98.0	2.0	0	0
nox	0	0	0	4.0	96.0	0	0
美	0	0	0	2.0	0	94.0	4.0
游	0	0	0	0	0	2.0	98.0

**Table 13:** Accuracy comparison among the MGRA, best-uWave, 10-uWave and 6DMG test static model with static and mobile traces on Confusion Set and Easy Set.

Gesture Sets	Static				Mobile			
	MGRA	Best-uWave	10-uWave	6DMG	MGRA	Best-uWave	10-uWavw	6DMG
Confusion Set	95.83%	89.13%	89.87%	91.32%	89.92%	72.46%	72.90%	73.89%
Easy Set	98.70%	92.46%	93.31%	95.79%	95.40%	82.42%	83.76%	85.14%

**Table 14:** Comparison of online time consumption and energy consumption among MGRA, 6DMG and uWave.

Approach	Training Time (s)	Classification Time (s)	Training Energy (J)	Classification Energy (J)
MGRA	54.494	0.161	26.600	0.396
best-uWave	34.533	0.376	39.271	0.478
10-uWave		3.840		4.892
6DMG	112.443	0.298	128.700	0.829
